# Intentional vomiting as a rare cause of hypercalcemia and consequent acute renal failure: a case report

**DOI:** 10.3389/fmed.2024.1394601

**Published:** 2024-06-28

**Authors:** Mihovil Santini, Ana Sorić, Pavao Mioč, Siniša Car, Kristijan Đula, Ivan Zeljkovic

**Affiliations:** ^1^General Hospital Zadar, Zadar, Croatia; ^2^Health Center Zagreb – West, Zagreb, Croatia; ^3^Sisters of Charity Hospital, Zagreb, Croatia; ^4^University Hospital Dubrava, Zagreb, Croatia

**Keywords:** acute renal failure, bulimia, dehydration, hypercalcemia, vomiting

## Abstract

Two most common causes of elevated serum calcium levels, which together account for nearly 90% of all cases, are primary hyperparathyroidism and malignancy. Thus, it is necessary to consider other disorders in the diagnostic evaluation of patients with hypercalcemia. We report the case of a 40-year-old female patient with an intellectual disability who was admitted to the Emergency Department with severe symptomatic hypercalcemia and acute renal failure, caused by recurrent intentional vomiting. The aim of this report is to help clinicians make an accurate diagnosis by considering recurrent vomiting habits as a potential cause of hypercalcemia and acute renal failure. Our case provides a comprehensive diagnostic work-up and multidisciplinary treatment strategies for patients with symptomatic hypercalcemia.

## Introduction

The two most common causes of elevated serum calcium levels, which together account for nearly 90% of all cases, are primary hyperparathyroidism and malignancy. The remaining 10% represent an important figure, and therefore it is necessary to consider other disorders in the evaluation of patients with hypercalcemia (1). Other less common causes of hypercalcemia are Milk-alkali syndrome, tertiary hyperparathyroidism, granulomatous diseases, thyrotoxicosis, drugs ingestion, immobilization and some inherited disorders (2–7). The incidence of hypercalcemia is about 1% worldwide (8). It can be classified into mild (<3 mmol/L), moderate (3–3.5 mmol/L) and severe (>3.5 mmol/L). Symptoms can vary from asymptomatic hypercalcemia to nausea, constipation, fatigue, dehydration, confusion, polyuria, polydipsia, somnolence, or even coma (8, 9). The relationship between hypercalcemia and renal failure is frequently reported in the medical literature, although the pathogenetic mechanisms are still unclear (10). Acute renal failure (ARF) in a patient with hypercalcemia most likely occurs because increased calcium concentration causes tubular damage and vasoconstriction of the afferent renal arteriole (2, 10). We report the case of a 40-year-old female patient with an intellectual disability who was admitted to the Emergency Department (ED) with severe symptomatic hypercalcaemia and ARF. The aim of this report is to help clinicians make an accurate diagnosis by considering recurrent vomiting habits as a potential cause of hypercalcemia and ARF.

## Case presentation

**Figure 1:**
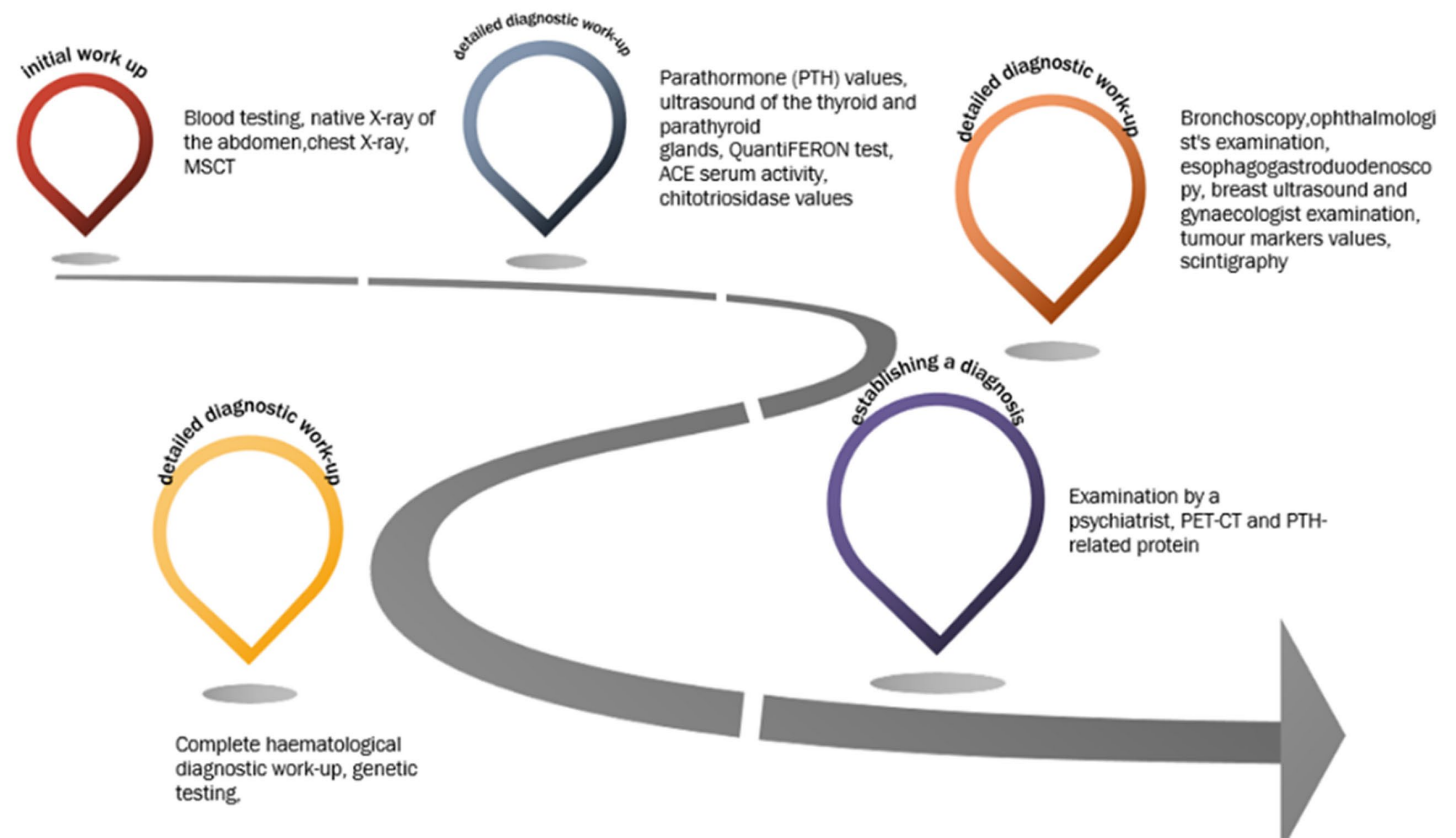
Timetable of the diagnostic work-up.

We present the case of a 40-year-old female patient with an intellectual disability, who was admitted to the ED due to nausea, fatigue, fever and abdominal pain. Abdominal pain began 7 days earlier, followed by repeated episodes of headache and vomiting. She complained of loss of appetite, constipation and polyuria. The patient had no comorbidities apart from intellectual disability which has been observed since childhood, but the diagnostic work-up and close follow-up was never made. On admission, she was febrile (up to 38 Celsius) with heart rate of 112 beats per minute, blood pressure 150/70 mmHg, respiratory rate of 18 breaths per minute, and oxygen saturation 98% on room air. Results of blood testing at admission, which should be highlighted, are shown in Table 1. Urinalysis showed sub-nephrotic proteinuria with initial signs of urinary tract infection (leukocyte esterase 1+, nitrite negative). Native X-ray of the abdomen ruled out acute abdomen but showed significant intestinal flatulence with coprostasis and dilatation of the transverse colon up to 6.5 cm in diameter. A chest X-ray showed no signs of inflammation. Abdominal and pelvic multi-slice computed tomography (MSCT) showed dilatation of ascending and transverse colon up to 6.5 cm with no signs of ileus or acute abdomen. Chest MSCT showed lung infiltrate and pleural effusion with a diffuse ground-glass opacities with thickening of the interlobular septa and mediastinal lymphadenopathy suggesting potential interstitial lung disease. After the initial diagnostic work-up was done, the patient was admitted to the intensive care unit (ICU) due to severe symptomatic hypercalcaemia and ARF combined with hyponatremia and hypokalaemia. In the ICU, correction of electrolyte imbalance and ARF was achieved with the use of zolendronic acid [4 mg intravenously (i.v.)] corticosteroids (prednisone in a dose of 20 mg/day) and crystalloid solutions (at an initial rate of 200–300 mL/h, then adjusted to maintain the urine output at 100–150 mL/h). Due to lung infiltrate and elevated inflammatory parameters, she was treated with intravenous administration of antibiotics (meropenem, ceftriaxone). Control laboratory findings showed the normalization of the electrolyte imbalance and a decrease in the values of inflammatory parameters (Table 1). After 7 days, the patient was discharged from the ICU and was transferred to the Endocrinology Department. Consequently, during and after hospitalization, detailed diagnostic work-up was performed to determine the aetiology of hypercalcemia. Parathormone (PTH) values were within the normal range (< 0.6 pmol/L), with ultrasound findings of the thyroid and parathyroid glands also normal, ruling out parathyroid pathology. A control chest MSCT (which was done at the time of discharge from the hospital) showed the complete regression of all the described changes, without signs of interstitial lung disease. QuantiFERON test was negative. No pathological accumulation of radiopharmaceuticals was observed by scintigraphy of the skeleton. The rheumatologist ruled out the systemic rheumatic diseases based on the control chest MSCT and laboratory findings. Sarcoidosis was ruled out by an ophthalmologist’s and pulmonologist’s examination [based on angiotensin-converting enzyme (ACE) serum activity, normal chitotriosidase values, bronchoscopy, chest MSCT and no ocular manifestation of sarcoidosis]. Esophagogastroduodenoscopy, breast ultrasound and gynaecologist examination combined with normal values of tumour markers ruled out a potential (para)neoplastic condition. A more detailed anamnesis (medical history), physical examination and endocrinological findings ruled out milk-alkali syndrome and the possibility of intoxication with vitamin D and ethylene glycol. Complete haematological diagnostic work-up (including bone marrow biopsy, cytological analysis and serum and urine protein electrophoresis) excluded multiple myeloma and other haematological diseases. Regarding to intellectual disability and single episode of hypercalcaemia, genetic testing was performed to rule out hereditary syndromes (i.e., tubulopathies) that can cause hypercalcemia, however the results did not reveal any genetic syndrome. As part of the treatment, the patient was examined by a psychiatrist who revealed the patient’s recurrent vomiting habits, most likely as part of bulimia after which psychotherapy was recommended. Our patient underwent psychotherapy and nutritional rehabilitation. Cognitive-behavioural therapy is the first-line treatment, however due to patient’s intellectual disability, the application of therapy was demanding, but feasible, with a more strict observation of her daily routine provided by her family. Considering that PET-CT and PTH-related protein, which were subsequently done later, definitively ruled out a (para)neoplastic condition, and all other findings were normal, the only explanation is that hypercalcaemia and consequent ARF were caused by repeated and excessive vomiting (see Figure 1).

**Table 1:** Patient’s laboratory findings throughout hospitalization.

Results of blood testing	At admission	Control laboratory – highlighted	Reference range
Haemoglobin	122 g/L	125 g/L	119–157 g/L
White blood cells	12.5 × 10^9^/L	5.9 × 10^9^/L	3.4–9.7 × 10^9^/L
Platelets	281 × 10^9^/L		158–424 × 10^9^/L
CRP	165.0 mg/L	2.6 mg/L	< 5 mg/L
ESR	50 mm/h		4–24 mm/h
Creatinine	232 μmol/L		49–90 μmol/L
Urea	19.9 mmol/L		2.8–8.3 mmol/L
eGFR	22.0 mL/min		>90 mL/min
AST	34 U/L		8–38 U/L
ALT	23 U/L		10–48 U/L
GGT	76 U/L		9–35 U/L
LDH	198 U/L		<241 U/L
Lipase	45 U/L		<67 U/L
RF	37 IU/mL		<14 IU/mL
Total serum calcium level	4.7 mmol/L	2.17 mmol/L	2.14–2.53 mmol/L
Ionised calcium	2.42 mmol/L	1.26 mmol/L	1.16–1.31 mmol/L
Sodium (Na)	130 mmol/L	142 mmol/L	137–146 mmol/L
Potassium (K)	3.0 mmol/L	4,6 mmol/L	3.9–5.1 mmol/L
Chloride (Cl)	88 mmol/L	106 mmol/L	97–108 mmol/L
Phosphate (P)	1.23 mmol/L	0.87 mmol/L	0.79–1.42 mmol/L
Magnesium (Mg)	0.73 mmol/L	0.75 mmol/L	0.79–1.42 mmol/L
TSH	0.48 mU/L		0.27–4.20 mU/L
FT4	17.7 pmol/L		11–22 pmol/L
FT3	3.28 pmol/L		3.95–6.8 pmol/L
PTH	<0.6 pmol/L		1.6–6.9 pmol/L

## Discussion

We described a rare case of hypercalcemia and ARF caused by bulimic behaviour in a patient with an intellectual disability. The causes of hypercalcemia can be numerous (1–7). Sometimes the aetiology of hypercalcemia can be found out from a detailed anamnesis (family history, use of supplements/drugs, etc.), but sometimes, as in our case, it is necessary to do a wide diagnostic work-up to exclude all potential causes.

The two main categories of mechanisms linked to hypercalcaemia are those mediated by PTH (primary and tertiary hyperparathyroidism, familial benign hypocalciuric hypercalcaemia, Lithium induced hypercalcaemia, etc.) and those mediated by non-PTH (associated to production of calcitriol, PTH related protein, or cytokines as mediators) (1). In general, primary (and occasionally tertiary) hyperparathyroidism is the most common cause of hypercalcaemia if PTH is high. On the other hand, if the PTH level is decreased, malignancy is most likely the cause of hypercalcaemia, however other PTH-independent disorders should also be investigated (3). Numerous electrolyte abnormalities, such as hypokalaemia, hyponatraemia, hypomagnesaemia, hypophosphatemia, and hypocalcaemia have been linked to eating disorders. Eating disorders contribute substantially to wide-spread organ dysfunction and ARF is one of them. There have been reports of hypercalcaemia associated with disordered eating as a result of milk-alkali syndrome, dehydration, or thiazide diuretic abuse where patients suffering from bulimia were particularly vulnerable. In the setting of hypo- and hypercalcemia, nephrocalcinosis and renal impairment have been reported in eating disorder patients. Nephrocalcinosis occurs as a result of chronic volume loss, negative calcium balance (due to phosphate depletion and bone resorption) and progressive hypercalciuria. Also, aldosterone directly affects urinary calcium excretion. To establish correct diagnosis, all other causes of hypercalcaemia should be excluded before attributing it to disordered eating (11, 12). Our patient has suffered ARF due to a combination of dehydration and hypercalcaemia. After all other causes have been definitively ruled out, the only explanation is that hypercalcaemia was caused by an excessive vomiting/bulimia. Identifying and treating the underlying cause of hypercalcemia is the most crucial stage in the treatment process. The cornerstone of hypercalcemia treatment is hydration (0.9% saline intravenously, starting with 1–2 L) and bisphosphonates (pamidronate and zoledronic acid). Corticosteroids are limited to the treatment of malignancy-related hypercalcemia while denosumab is approved for malignancy-related hypercalcemia that is unresponsive to bisphosphonates (13–15).

## Conclusion

Except emphasizing the significance of considering bulimic behaviour in patients presenting with hypercalcaemia, this case emphasizes the importance of taking a detailed and repeated anamnesis/heteroanamnesis, which is sometimes the most useful diagnostic method to determine the aetiology of hypercalcemia.

## Data availability statement

The original contributions presented in the study are included in the article/supplementary material, further inquiries can be directed to the corresponding author.

## Ethics statement

Written informed consent was obtained from the individual(s) for the publication of any potentially identifiable images or data included in this article.

## Author contributions

MS: Writing – original draft, Writing – review & editing. AS: Writing – original draft, Writing – review & editing. PM: Writing – original draft, Writing – review & editing. SC: Writing – original draft, Writing – review & editing. KĐ: Writing – original draft, Writing – review & editing. IZ: Writing – original draft, Writing – review & editing.
